# A high-throughput pipeline for detecting locus-specific polymorphism in hexaploid wheat (*Triticum aestivum* L.)

**DOI:** 10.1186/s13007-015-0082-6

**Published:** 2015-08-04

**Authors:** Jian Ma, Jiri Stiller, Zhi Zheng, Ya-Xi Liu, Yuming Wei, You-Liang Zheng, Chunji Liu

**Affiliations:** Triticeae Research Institute, Sichuan Agricultural University, Wenjiang, Chengdu, 611130 China; CSIRO Agriculture Flagship, 306 Carmody Road, St Lucia, QLD 4067 Australia; School of Plant Biology, The University of Western Australia, Perth, WA 6009 Australia; National Foxtail Millet Improvement Centre, Institute of Millet Crops, Hebei Academy of Agricultural and Forestry Sciences, Shijiazhuang, China

**Keywords:** Allopolyploid, Multiple sequence alignment, blastn, Primer design, Genome-specificity, Sequence polymorphism

## Abstract

**Background:**

Bread wheat (*Triticum aestivum* L., 2n = 6x = 42) is an allohexaploid with a huge genome. Due to the presence of extensive homoeologs and paralogs, generating locus-specific sequences can be challenging, especially when a large number of sequences are required. Traditional methods of generating locus-specific sequences are rather strenuous and time-consuming if large numbers of sequences are to be handled.

**Results:**

To improve the efficiency of isolating sequences for targeted loci, a time-saving and high-throughput pipeline integrating orthologous sequence alignment, genomic sequence retrieving, and multiple sequence alignment was developed. This pipeline was successfully employed in retrieving and aligning homoeologous sequences and 83% of the primers designed based on the pipeline successfully amplified fragments from the targeted subgenomes.

**Conclusions:**

The high-throughput pipeline developed in this study makes it feasible to efficiently identify locus-specific sequences for large numbers of sequences. It could find applications in all research projects where locus-specific sequences are required. In addition to generating locus-specific markers, the pipeline was also used in our laboratory to identify differentially expressed genes among the three subgenomes of bread wheat. Importantly, the pipeline is not only valuable for research in wheat but should also be applicable to other allopolyploid species.

**Electronic supplementary material:**

The online version of this article (doi:10.1186/s13007-015-0082-6) contains supplementary material, which is available to authorized users.

## Background

Reference genome sequences of several major crops have been reported and include rice [1], barley [2], foxtail millet [3], maize [4], sorghum [5], potato [6], tomato [7] and *Brassica napus* [8]. Significant progress has also been made in recent years in generating reference genomes for bread wheat [9] and its progenitor species [10, 11]. These genome sequences have been extensively exploited in the whole spectrum of biological studies ranging from basic understanding of crop evolution to applied breeding. With the rapid development in sequencing capacity, it is anticipated that whole genome sequences should soon become available for multiple genotypes for each of the species of agronomic importance.

Knowing the origins of specific DNA or RNA sequences is essential in numerous applications, such as designing locus-specific markers. Although gene duplication is a common feature of all plant species including *Brachypodium* [12], rice [1], and barley [2], developing locus-specific markers for these diploid species is relatively easy. This suggests that enough variation must exist between the majority of duplicated genes in these species. However, isolating locus-specific sequences for a given subgenome of interest from bread wheat or other polyploid species is still challenging as two or more homoeologous sequences exist in each of these genomes. It can be even more daunting when isolating a gene of interest which belongs to an orthologous gene set or a gene family.

Currently several steps need to be taken when isolating a specific homoeologous sequence from an allopolyploid species. First, web-based blast servers such as National Center for Biotechnology Information (NCBI) or ViroBLAST in Unité de Recherche Génomique Info (URGI, https://urgi.versailles.inra.fr/blast/blast.php) [13] need to be employed to search for orthologous sequences for a given query sequence. Second, all the orthologous gene sequences for a given species need to be manually retrieved from contigs or scaffold sequences. Third, multiple sequence alignment tools are required to align retrieved orthologous sequences to detect locus-specific sequences. This procedure can be used to manage a limited number of sequences but will become rather strenuous and time-consuming if large numbers of sequences need to be handled.

To improve the efficiency of retrieving sequences from polyploidy species, we have developed a pipeline by streamlining the steps in orthologous sequence alignment, genomic sequence retrieving and multiple sequence alignment. This time-saving and high-throughput pipeline significantly simplifies the detection of locus-specific sequences in allopolyploid species. The pipeline has also been successfully used in differentiating expressed genes among the three bread wheat subgenomes.

## Results and discussion

**Fig. 1:**
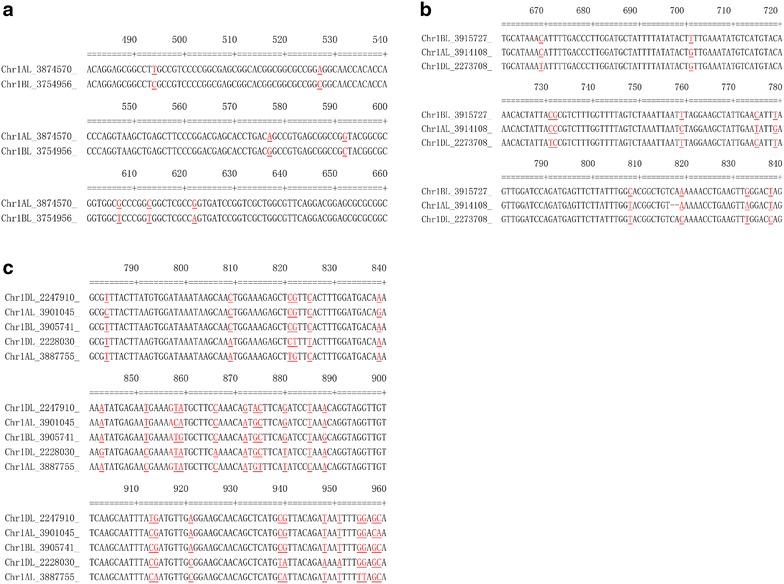
Examples of multiple alignments for two (**a**), three (**b**), and five (**c**) homoeologous sequences for identifying allele-specific sequences for marker development.

The percentages of query gene sequences which detected two or more orthologous sequences from chromosome shotgun sequences (CSSs) were about 90% from *Brachypodium*, 93% from *Ae. tauschii* and 95% from *T. urartu* (Table 1). Examples of these stringent alignments containing two orthologous sequences (e.g. *Bradi2g16370.1*), three homoelogous sequences (e.g. *Bradi2g33190.1*), and more than four homoeologous sequences (e.g. *Bradi2g14840.1*) are shown in Fig. 1. Each of the alignments with the suffix ‘.htm’ is easily readable by any web browser. The alignments generated and described in ‘Methods’ can be directly used to check possible allele-specific loci for isolating genes in hexaploid wheat.

**Table 1:** Numbers of genes used in blasting against wheat chromosome shotgun sequences (CSSs)

	*Brachypodium distachyon*	*Triticum urartu*	*Aegilops tauschii*
Total numbers of genes	31,029	34,879	43,150
Genes with hits on CSSs	30,028	34,671	43,126
Genes with generated alignments	27,782	32,378	40,961

**Fig. 2:**
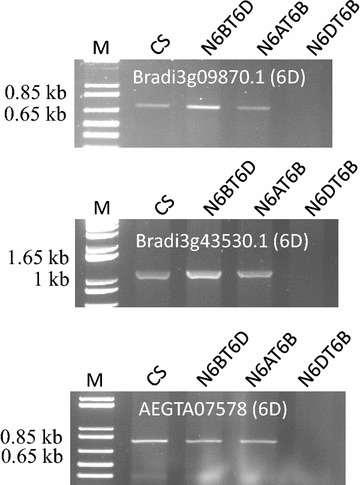
PCR profiles of the hexaploid wheat genotype ‘Chinese Spring’ and its nulli-tetrasomic (NT) lines with primers for three genes assessed. Locations of the genes detected from the chromosome shotgun sequences are given in *brackets*. 1 kb plus DNA ladder (M) was used as the size marker.

**Fig. 3:**
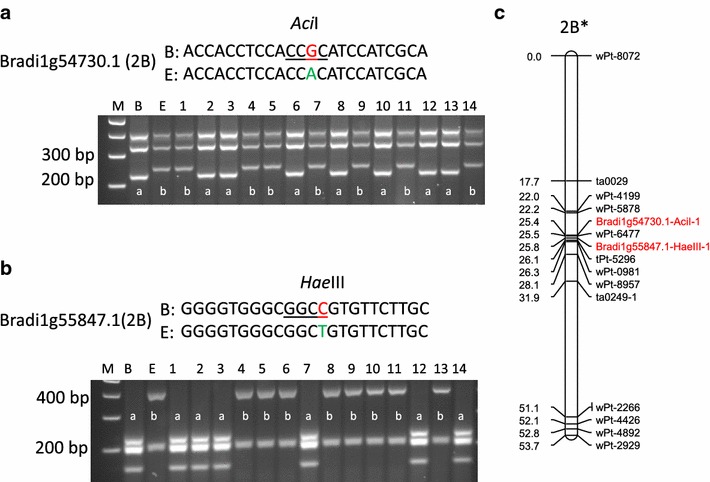
Validation of marker locations using a DH (doubled haploid) population. Orthologous sequences of *Bradi1g54730.1* and *Bradi1g55847.1* were amplified from the two parents of the DH population, B (‘Batavia’) and E (‘Ernie’), and sequenced. The single nucleotide polymorphism (in *red* and *green*) and restriction enzyme sites (*underlined*) were identified between B and E for *Bradi1g54730.1* with restriction enzyme *Aci*I (**a**) and *Bradi1g55847.1* with *Hae*III (**b**). The amplified products of the two parents and 14 of the DH lines were digested and separated on agarose gels. Map positions of the two new markers on chromosome 2B (**c**) were calculated based on the linkage map published by Li et al. [21].

Given that primers designed based on a single nucleotide polymorphisms (SNP) did not always amplify a specific fragment in our previous studies, primers designed in this study were based on two or more SNPs or indels (Additional file 1: Table S1). Of the 36 primer pairs designed for selected loci, 30 (83%) amplified a product on the expected chromosomes, two failed to amplify any PCR products, and the other four generated locus-specific fragments (Fig. 2 and Additional file 1: Table S1). Eleven of the 30 pairs of primers were further assessed against other bread wheat genotypes (Additional file 1: Table S1). Sequence alignments indicated that, without exception, they all amplified sequences homologous with those from the expected chromosomes as shown in ‘Chinese Spring’ (‘CS’) (Additional file 1: Table S1). Four of these primer pairs generated polymorphic fragments between the parents of the mapping population used in this study. The polymorphic sequences were used to develop cleaved amplified polymorphic sequence (CAPS) markers. Each of the four CAPS markers was successfully mapped to the anticipated chromosome as originally detected using ‘CS’ aneuploids (Fig. 3, Additional file 2: Fig. S1 and Additional file 3: Fig. S2).

A pipeline for generating SNP markers, PolyMarker, was reported recently and it is used to design primers for KASP™ (Kompetitive Allele Specific PCR) assay. KASP™ is a very unique system in that it uses three primers in PCR reactions. Two of them are allele-specific forward primers. Sequences from parental genotypes are required in designing the two forward primers which make accurate bi-allelic discrimination possible [14–18].

**Fig. 4:**
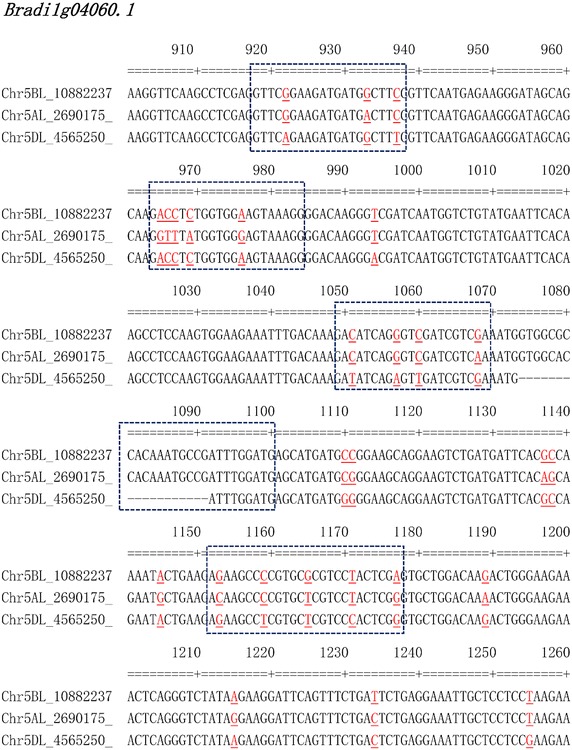
An example of selecting primer sequences for qPCR analysis. *Dotted boxes* represent regions that could be used for primer design.

Different from the PolyMarker/KASPTM system, the method reported in this paper does not need sequences from parental genotypes. Allele-specific primers are designed based on sequence alignments from all subgenomes of a species. In addition to designing allele-specific markers as shown in the current study, we have also adapted the pipeline to design allele-specific primers for reverse transcription quantitative PCR (RT-qPCR) analysis in wheat. For example, several positions of orthologous sequences of *Bradi1g04060* could be used to design RT-qPCR primers in bread wheat (Fig. 4). We have also successfully used the pipeline to retrieve conserved regions that could be used for differentially expressed analysis in bread wheat (not published). Obviously, this high-throughput pipeline would be applicable to other allopolyploid species such as rapeseed, cotton, or oat.

## Conclusion

Here we reported on a high-throughput pipeline which integrates orthologous sequence alignment, genomic sequences retrieval, and multiple sequence alignment. The pipeline can be conveniently used to identify locus-specific sequences for marker development and RT-qPCR and transcriptome analyses, especially when large numbers of sequences need to be dealt with. Examples of its application in wheat are given in this publication but the pipeline would also be valuable for similar applications in other allopolyploid species as well.

## Methods

### Plant materials

The euploid and selected nullisomic-tetrasomic ‘CS’ lines [19, 20] were used to locate PCR-amplified fragments to specific chromosomes. Two wheat populations were employed to further validate the location of DNA fragments amplified from primers designed in this study. One is a doubled haploid (DH) population with 153 lines generated from the ‘Batavia’/’Ernie’ cross [21], and the other one is an F8 population of recombinant inbred lines (RILs) with 92 lines derived from the ‘Lang’/‘CSCR6’ cross [22].

### Data collection

Gene-coding sequences (CDS) of *Brachypodium* genome version 1.2 were downloaded from http://www.plantgdb.org/BdGDB [12]. CDS of *Ae. tauschii* (wheatD_final_43150.gff.cds) [10] and *T. urartu* (TRIUR3_120813_filter150_cds) [11] were both downloaded from GIGA_DB (http://gigadb.org/). CSSs of ‘CS’ were downloaded from https://urgi.versailles.inra.fr/download/iwgsc/Science/ [9].

### Generation of multiple sequence alignments and primer design

**Fig. 5:**
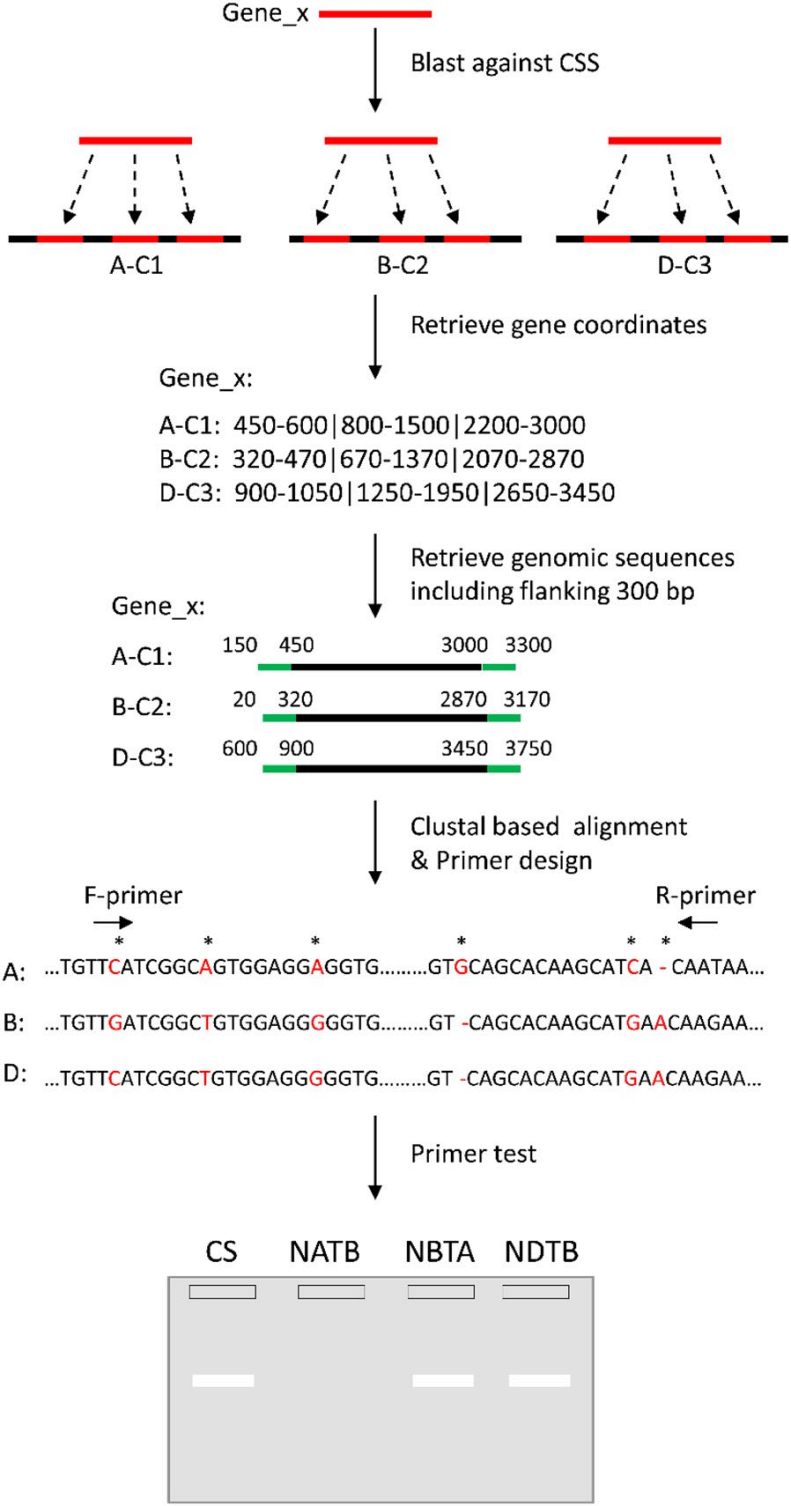
The pipeline of generating multiple sequence alignments in bread wheat. *CSSs* chromosome shotgun sequences; *A*, *B*, and *D* the three subgenomes of bread wheat; *C1*, *C2*, and *C3* the three contigs from the three subgenomes; ‘*CS*’ euploid wheat ‘Chinese Spring’; *NATB*, *NBTA*, and *NDTB* 3 nulli-tetrasomic (NT) lines for chromosomes belonging to a given homoeologous group. *Asterisks* representing polymorphic bases targeted in designing primers specific to a subgenome. The lack of a PCR product in the lane of nulli-A and tetra-B (or NATB) indicates that primers are specific to chromosome A.

Alignments of orthologous sequences from *Brachypodium*, *Ae. tauschii*, and *T. urartu* were generated following the steps outlined in Fig. 5. First, gene sequences from *Brachypodium*, *Ae. tauschii*, and *T. urartu* were blasted against CSSs using the BLAST + blastn algorithm with the parameters ‘-num_descriptions 10 -num_alignments 10 -evalue 0.00001’ (i.e. a maximum of 10 hits for each gene query and with E-value threshold of 10^−5^) [23]. Second, an in-house script was used to retrieve the coordinates of each hit for a given gene query from the blast results. A maximum of 5,000 bp intron and minimum of 200 bp exon were used to limit the retrieved coordinates for a given hit. Third, the 5′ and 3′ flanking regions of 300 bp were isolated from each of the contigs (hits) according to the coordinates obtained. Fourth, the isolated genomic sequences from all the hits for a given query were written to a single file. Finally, a script integrated with Gblocks_0.91ba [24] and Clustal W 2.1 [25] was used to generate the alignments of all the retrieved genomic sequences for a given query (Fig. 5). The alignments and in-house developed scripts are available at http://dx.doi.org/10.6084/m9.figshare.1393103; http://dx.doi.org/10.6084/m9.figshare.1393106; http://dx.doi.org/10.6084/m9.figshare.1393105.

### Validation of primers designed from the alignments

For validating the efficiency of the multiple sequence alignments generated, genes that were polymorphic between the parental lines of the mapping populations were assessed. Where possible, sequences differing in more than 1 SNP or indel were used to design primers targeting specific chromosomes for the selected genes (Additional file 1: Table S1).

The euploid and selected nullisomic–tetrasomic lines of ‘CS’ [19, 20] were analysed. Genomic DNA was extracted from 20-day-old seedlings using the hexadecyltrimethylammonium bromide method (CTAB) [26]. PCR amplification was performed in 10 μl reaction mixtures with 50 ng of genomic DNA, 200 μM of each dNTP, 0.2 μM of each primer, and 0.5 units of *Taq* polymerase. The cycling parameters were 94°C for 5 min to pre-denature, which was followed by 35 cycles of 94°C for 45 s, 40 s at the appropriate annealing temperature (ranging from 50 to 70°C depending on the primers, see Additional file 1: Table S1), 72°C for 1 min, and a final extension at 72°C for 10 min. Amplification products were separated on 1.5% agarose gels.

To further confirm the effectiveness of the primers in the RIL and DH populations, fragments of interest were purified using the QIAquick Extraction Kit (QIAGEN). The recovered PCR fragments were inserted into the pGEM-T easy vector (Promega) and transformed into *Escherichia coli* (Top10). At least three independent clones for each fragment were sequenced in both directions by the Australian Genome Research Facility Ltd. Sequenced fragments were aligned using by the DNAman software package (V5. 2.10; Lynnon Biosoft). To identify whether the sequenced fragments were from the expected chromosomes as found in the ‘CS’ aneuploids, they were aligned with all of the orthologous sequences from ‘CS’ for a given gene. SNPs between the parents of a given population were exploited to develop cleaved amplified polymorphic sequence (CAPS) marker using dCAPS Finder 2.0 [27]. PCR products were digested with appropriate enzymes from New England Biolabs (NEB) based on target sequences differences and separated on 3% agarose gels. The genetic linkage map was generated using JoinMap 4 [28].

## Additional files

Additional file 1: Table S1. Details of primers tested. Additional file 2: Figure S1. Validation of marker location of *AEGTA18760* using a RIL (recombination inbred lines) population. Orthologous sequences of *AEGTA18760* were amplified from the two parents of the RIL population, C (‘CSCR6’) and L (‘Lang’), and sequenced. The single nucleotide polymorphism (in red and green) and restriction enzyme sites (underlined) were identified between C and L for *AEGTA18760* with restriction enzyme *NlaIII* (A). The amplified products of the two parents and 13 of the RIL lines were digested and separated on agarose gels. The map position of the new marker on chromosome 3B (B) was calculated based on the linkage map published by Ma et al. [22]. Additional file 3: Figure S2. Validation of marker location of *Bradi1g07500*.1 using a DH (doubled haploid) population. Orthologous sequences of *Bradi1g07500*.1 were amplified from the two parents of the DH population, B (‘Batavia’) and E (‘Ernie’), and sequenced. The single nucleotide polymorphism (in red and green) and restriction enzyme sites (underlined) were identified between B and E for *Bradi1g07500*.1 with restriction enzyme *BtgI* (A). The amplified products of the two parents and 13 of the RIL lines were digested and separated on agarose gels. The map position of the new marker on chromosome 3B (B) was calculated based on the linkage map published by Li et al. [21].

## Abbreviations

CS: Chinese spring
DH: doubled haploid
RILs: recombinant inbred lines
CDS: coding sequences
CSSs: chromosome shotgun sequences
CAPS: cleaved amplified polymorphic sequence
CTAB: hexadecyltrimethylammonium bromide
SNP: single nucleotide polymorphism
RT-qPCR: reverse transcription quantitative PCR
NCBI: National Center for Biotechnology Information
