# Supplementary material for: A high-throughput pipeline for detecting locus-specific polymorphism in hexaploid wheat (Triticum aestivum L.)
Source: Plant Methods. 2015 Aug 4;11:39. doi: 10.1186/s13007-015-0082-6 (PMC4524443; doi:10.1186/s13007-015-0082-6)
Supplement: Additional file 2: Figure S1. — Validation of marker location of AEGTA18760 using a RIL (recombination inbred lines) population. Orthologous sequences of AEGTA18760 were amplified from the two parents of the RIL population, C (‘CSCR6’) and L (‘Lang’), and sequenced. The single nucleotide polymorphism (in red and green) and restriction enzyme sites (underlined) were identified between C and L for AEGTA18760 with restriction enzyme NlaIII (A). The amplified products of the two parents and 13 of the RIL lines were digested and separated on agarose gels. The map position of the new marker on chromosome 3B (B) was calculated based on the linkage map published by Ma et al. [22]. [file 13007_2015_82_MOESM2_ESM.pptx]

## Slide 1
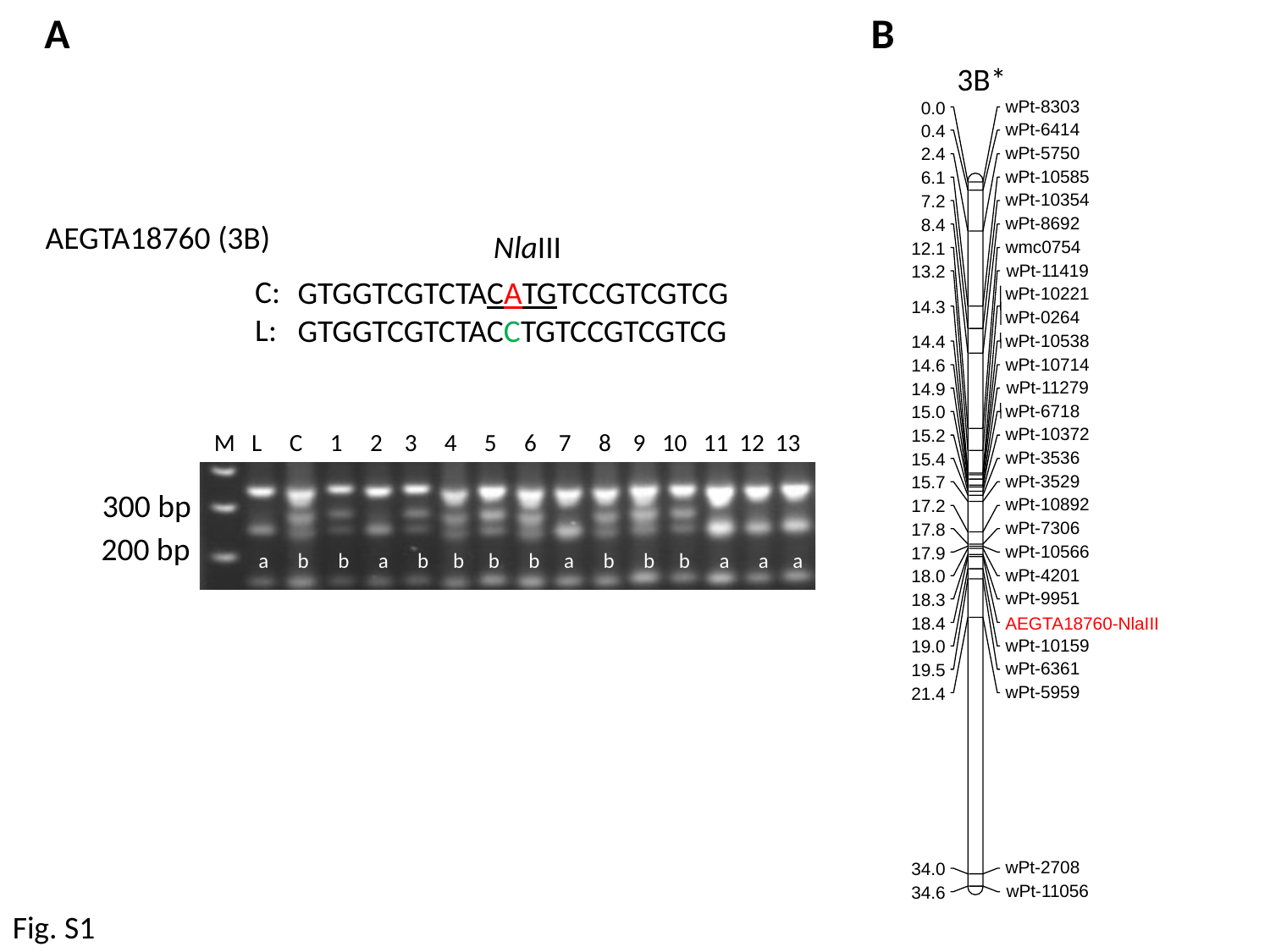

A
B
wPt-8303
0.0
wPt-6414
0.4
wPt-5750
2.4
wPt-10585
6.1
wPt-10354
7.2
wPt-8692
8.4
wmc0754
12.1
wPt-11419
13.2
wPt-10221
14.3
wPt-0264
wPt-10538
14.4
wPt-10714
14.6
wPt-11279
14.9
wPt-6718
15.0
wPt-10372
15.2
wPt-3536
15.4
wPt-3529
15.7
wPt-10892
17.2
wPt-7306
17.8
wPt-10566
17.9
wPt-4201
18.0
wPt-9951
18.3
18.4
AEGTA18760-NlaIII
wPt-10159
19.0
wPt-6361
19.5
wPt-5959
21.4
wPt-2708
34.0
wPt-11056
34.6
AEGTA18760 (3B)
NlaIII
C:
L:
GTGGTCGTCTACATGTCCGTCGTCG
GTGGTCGTCTACCTGTCCGTCGTCG
M L C 1 2 3 4 5 6 7 8 9 10 11 12 13
300 bp
200 bp
3B*
 a b b a b b b b a b b b a a a
Fig. S1
