# Supplementary material for: A high-throughput pipeline for detecting locus-specific polymorphism in hexaploid wheat (Triticum aestivum L.)
Source: Plant Methods. 2015 Aug 4;11:39. doi: 10.1186/s13007-015-0082-6 (PMC4524443; doi:10.1186/s13007-015-0082-6)
Supplement: Additional file 3: Figure S2. — Validation of marker location of Bradi1g07500.1 using a DH (doubled haploid) population. Orthologous sequences of Bradi1g07500.1 were amplified from the two parents of the DH population, B (‘Batavia’) and E (‘Ernie’), and sequenced. The single nucleotide polymorphism (in red and green) and restriction enzyme sites (underlined) were identified between B and E for Bradi1g07500.1 with restriction enzyme BtgI (A). The amplified products of the two parents and 13 of the RIL lines were digested and separated on agarose gels. The map position of the new marker on chromosome 3B (B) was calculated based on the linkage map published by Li et al. [21]. [file 13007_2015_82_MOESM3_ESM.pptx]

## Slide 1
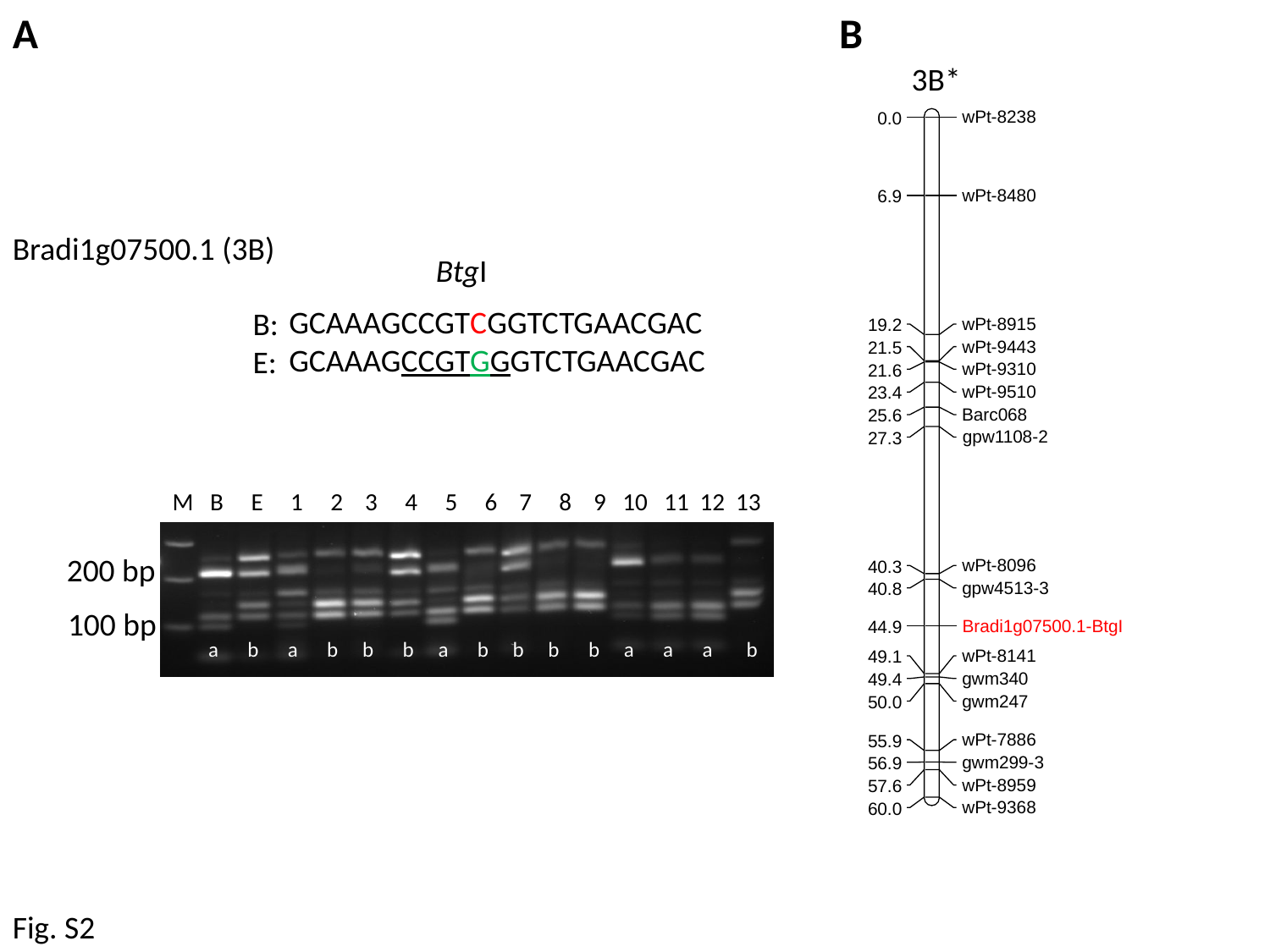

A
B
wPt-8238
0.0
wPt-8480
6.9
wPt-8915
19.2
wPt-9443
21.5
wPt-9310
21.6
wPt-9510
23.4
Barc068
25.6
gpw1108-2
27.3
wPt-8096
40.3
gpw4513-3
40.8
Bradi1g07500.1-BtgI
44.9
wPt-8141
49.1
gwm340
49.4
gwm247
50.0
wPt-7886
55.9
gwm299-3
56.9
wPt-8959
57.6
wPt-9368
60.0
Bradi1g07500.1 (3B)
BtgI
GCAAAGCCGTCGGTCTGAACGAC
GCAAAGCCGTGGGTCTGAACGAC
B:
E:
M B E 1 2 3 4 5 6 7 8 9 10 11 12 13
200 bp
100 bp
3B*
 a b a b b b a b b b b a a a b
Fig. S2
